# Microwave Pre-Treatment for Efficient Zinc Recovery via Acid Leaching

**DOI:** 10.3390/ma18112496

**Published:** 2025-05-26

**Authors:** Bagdaulet Kenzhaliyev, Ainur Berkinbayeva, Kenzhegali Smailov, Zhazira Baltabekova, Shynar Saulebekkyzy, Nazerke Tolegenova, Azamat Yessengaziyev, Nauryzbek Bakhytuly, Symbat Tugambay

**Affiliations:** The Institute of Metallurgy and Ore Beneficiation, Satbayev University, Almaty 050013, Kazakhstan; bagdaulet_k@satbayev.university (B.K.); a.n.berkinbayeva@satbayev.university (A.B.); zh.baltabekova@satbayev.university (Z.B.); sh.saulebekkyzy@satbayev.university (S.S.); n.tolegenova@satbayev.university (N.T.); a.yessengaziyev@satbayev.university (A.Y.); n.bakhytuly@satbayev.university (N.B.); 970531400857-d@satbayev.university (S.T.)

**Keywords:** microwave processing, zinc extraction, phase transformations, acid leaching, hydrometallurgy, zinc clinker

## Abstract

This study presents an innovative approach to processing refractory zinc-bearing clinker using microwave thermal treatment followed by acid leaching. Microwave irradiation induces phase transformations, converting sphalerite (ZnS) to zincite (ZnO), and generates microcracks that enhance clinker porosity and reactivity. These changes significantly improve zinc dissolution during sulfuric acid leaching. Key parameters—acid concentration, temperature, solid-to-liquid ratio, and leaching time—were optimized, achieving a zinc extraction of 92.5% under optimal conditions (40 g/L H_2_SO_4_, solid-to-liquid ratio 1:4, 600 °C, 5–7 min) compared to 39.1% without pre-treatment. Thermodynamic analysis confirms the higher reactivity of ZnO, driven by favorable Gibbs free energy and exothermic reaction characteristics. These findings demonstrate the potential of microwave processing to intensify hydrometallurgical processes, offering energy efficiency and environmental benefits for industrial zinc recovery.

## 1. Introduction

Zinc, as a non-ferrous metal, plays a pivotal role in various industrial applications due to its unique combination of physicochemical properties, including outstanding corrosion resistance, galvanic protection, and catalytic versatility [1,2,3,4]. Its indispensability is evident across critical sectors: in construction, where it enhances the durability of steel structures through galvanization [5]; in the automotive industry, as a key component in corrosion-resistant parts; in electronics, where it is used in essential conductive and protective elements; and in pharmaceuticals, where its compounds are integral to antimicrobial and wound-healing formulations. Moreover, zinc is fundamental in energy storage technologies, including alkaline and zinc-air batteries [6], and serves as a key precursor in the synthesis of pigments, polymers, and other advanced chemical products [7]. Globally, zinc consumption ranks only behind iron, aluminum, and copper [8,9].

The depletion of high-grade zinc ores and the escalating global demand have shifted the focus toward unconventional sources, including technogenic waste, low-grade ores, and even marine and oceanic deposits, as viable alternatives for metal recovery [10,11,12,13,14,15]. Sulfide ores remain the predominant source of zinc, with flotation-based beneficiation historically ensuring stable supplies [16]. However, the growing global demand and the depletion of high-quality sulfide deposits have rendered reliance on primary resources increasingly unsustainable [17]. Secondary sources, such as metallurgical dust, electronic waste, and spent batteries, have become indispensable, now accounting for approximately 40% of recycled zinc production [18,19]. In industrially developed countries, the utilization rate of industrial waste reaches 70–80% [20,21], while in Kazakhstan, a major mineral producer, this rate remains low due to the lack of advanced technologies and equipment for waste processing [22]. This disparity underscores the urgent need for innovative solutions, particularly in regions like Kazakhstan, where an estimated 4.5 to 5.7 million tons of zinc industry waste has accumulated, exacerbating environmental and economic challenges [23]. The Commonwealth of Independent States (CIS) extracts around 3.5 billion cubic meters of solid minerals annually, generating approximately 1.5 billion cubic meters of waste, much of which is stockpiled in tailings facilities, further highlighting the scale of the challenge.

Zinc clinker, a byproduct of metallurgical operations, exemplifies the challenges associated with secondary resource utilization [24,25]. Containing industrially significant concentrations of valuable metals—zinc (0.8–3.6%), copper (0.6–4.1%), and lead (0.4–2.8%)—alongside substantial amounts of carbon (10–40%) and iron (15–35%), it presents considerable metallurgical complexity. Additionally, the presence of precious metals, including gold (1.0–10.0 g/t) and silver (100–750 g/t), highlights the untapped potential of this waste stream. Traditional pyrometallurgical approaches, such as Vanukov furnace smelting, while effective for metal extraction, are highly energy intensive (>1000 °C) and environmentally detrimental due to significant emissions [26]. These methods also result in substantial metal losses due to the volatility of zinc compounds and increased slag formation, with recovery rates typically ranging from 70% to 85% [27].

In response to increasingly stringent environmental regulations and the need to reduce energy consumption, hydrometallurgical processes based on acidic and alkaline leaching have gained widespread adoption [28,29,30]. Among leaching agents, sulfuric acid is the most widely used due to its high efficiency, cost-effectiveness, and broad availability [31]. Although acid leaching offers excellent metal selectivity and recovery, its efficiency is often hindered by the complex mineralogical composition of clinker. Alkaline leaching with agents such as NaOH and ammonia provides advantages in terms of equipment longevity and environmental safety; however, it is constrained by the high viscosity of residues and suboptimal recovery rates when processing low-grade raw materials [32]. While ammonia-based leaching mitigates some of the limitations associated with acid methods, it is compromised by ammonia volatility, leading to operational inefficiencies and increased costs.

Microwave (MW) irradiation has emerged as a transformative pre-treatment method that selectively disrupts refractory mineral matrices. The structural modifications induced by MW energy, manifested as weakened bonds between valuable minerals and gangue, enhance the material’s susceptibility to chemical treatment, thereby improving leaching kinetics and overall metal recovery. This selective dielectric heating facilitates phase transformations, such as the conversion of sphalerite (ZnS) to zinc oxide (ZnO), which significantly improves acid leachability, reduces energy consumption, and minimizes environmental impact [33,34,35,36]. MW heating combined with Na_2_O_2_ addition has been shown to achieve a zinc leaching rate of 82.06% from oxide–sulphide ores while avoiding SO_2_ emissions, offering an environmentally friendly alternative to traditional roasting [37]. MW processing also offers significant energy savings by targeting specific mineral phases, reducing waste volumes, and mitigating the environmental impact of waste disposal sites.

The present study focuses on investigating the phase transitions and structural transformations of zinc clinker under microwave irradiation, with particular emphasis on enhancing its reactivity with sulfuric acid. The objective of this work is to develop an energy-efficient and environmentally sustainable approach for zinc extraction from complex technogenic waste, thereby improving resource utilization and reducing environmental impact. The findings are expected to contribute to the development of a novel, highly efficient hydrometallurgical process for the extraction of valuable metals from complex refractory zinc-containing waste.

## 2. Materials and Methods

### 2.1. Materials

This study focused on a technogenic zinc-bearing material—clinker, characterized by complex processing challenges. X-ray fluorescence analysis revealed significant contents of iron (20.98%), calcium (5.27%), silicon (9.59%), oxygen (31.27%), copper (3.21%), zinc (3.47%), and other elements, as listed in Table 1.

### 2.2. Analytical Techniques

This research employed advanced analytical methodologies to examine the phase composition of clinker and to elucidate the underlying mechanisms governing phase transformations. Phase composition analysis was carried out using a Bruker D8 Advance X-ray diffractometer (Bruker, Ettlingen, Germany) under the following operational conditions: a scanning range of 4–90°, Cu–Kα radiation (λ = 0.15406 nm), an applied tube voltage of 40 kV, a tube current of 40 mA, and a continuous scanning rate of 1°/min. The elemental composition of the samples was determined using an Axios 1 kW wavelength-dispersive X-ray fluorescence spectrometer (PANalytical, Almelo, The Netherlands), with data acquisition and interpretation conducted via SuperQ5 software (Omnian 37). Surface microstructural characterization was performed using a JXA-8230 electron probe microanalyzer (JEOL, Tokyo, Japan) operating at an accelerating voltage of 20 kV, an electron beam current below 1 nA, and an aperture diaphragm No. 3. Additionally, energy-dispersive spectroscopy (EDS) microanalysis (JEOL, Tokyo, Japan) was conducted under conditions of an electron beam current up to 6 nA and a dead time reaching 14%. The quantification of zinc concentrations in liquid and solid-phase samples was carried out using an Optima 8300DV inductively coupled plasma atomic emission spectrometer (PerkinElmer, Inc., Waltham, MA, USA) along with a contrAA 800 atomic absorption spectrometer (Analytik Jena AG, Jena, Germany).

### 2.3. Experimental Method

**Figure 1:**
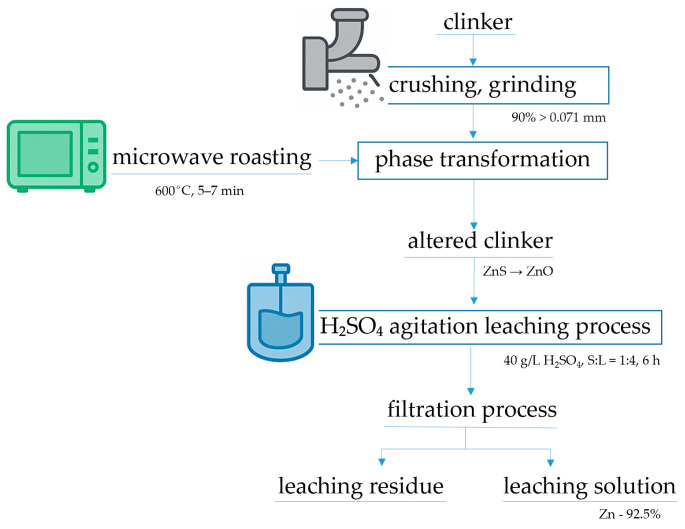
Schematic flow diagram of the experimental procedure.

**Figure 2:**
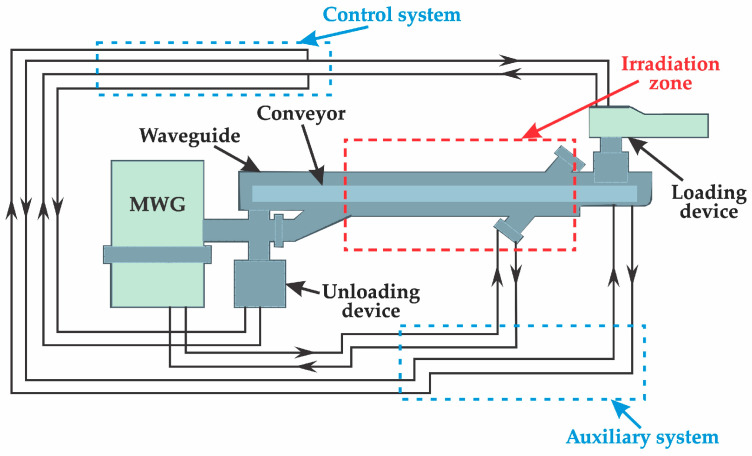
Schematic diagram of the “ENERGY K-50” microwave system.

**Figure 3:**
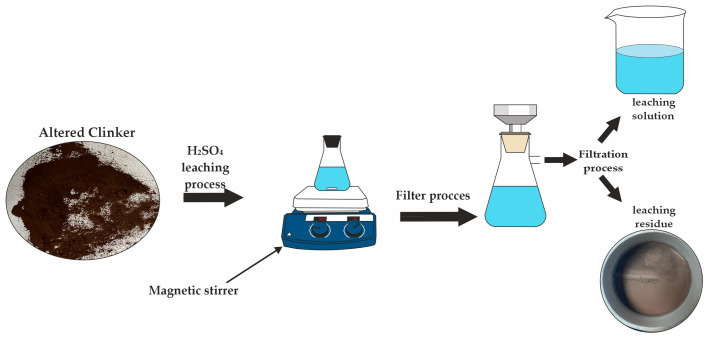
Schematic representation of the leaching experimental setup.

Zinc extraction from clinker was conducted in three key stages: grinding the raw material to a particle size where at least 90% of particles are below 0.071 mm, microwave-induced phase transformation, and subsequent acid leaching using sulfuric acid (H_2_SO_4_). A detailed schematic of the process sequence is presented in Figure 1, encompassing operations such as sample preparation and thermal treatment of clinker in a high-power microwave reactor “ENERGY K-50” (915 MHz, 25 kW) (Figure 2). This equipment is characterized by high productivity, operational stability, and enhanced efficiency [38,39,40,41,42]. The final stage—acid leaching—was performed in an H_2_SO_4_ solution under atmospheric pressure, as depicted in Figure 3.

The degree of zinc leaching was determined according to the following equation:

(1) $$\eta_{\mathrm{Zn}}=\left(\frac{\left(w_{\mathrm{clkr}.}\times m_{\mathrm{clkr}.}\;\right)-\left(w_{res}\times m_{res}\right)\;}{w_{\mathrm{clkr}.}\times m_{\mathrm{clkr}.}}\right)\times 100\%$$

where $\eta_{\mathrm{Zn}}$ represents the zinc leaching efficiency (%), $w_{\mathrm{clkr}.}$ is the zinc concentration in the initial clinker (%), $w_{res}$ is the zinc concentration in the solid residue after leaching (%), $m_{\mathrm{clkr}.}$ corresponds to the mass of the starting clinker sample (g), and $m_{res}$ denotes the mass of the leached residue (g). To ensure the reproducibility and accuracy of the results, each experimental trial was conducted at least three times, and the mean values obtained were utilized for further analysis.

The thermodynamic analysis of the sulfuric acid leaching process was conducted utilizing the HSC Chemistry 8.0 software (Metso Outotec, Espoo, Finland), employing its modules for reaction equations and Eh-pH diagrams.

To determine the thermodynamic functions characterizing individual substances, standard reference values of enthalpy ($H_{298}$), entropy ($S_{298}$), and polynomial coefficients (A, B, C, D) from the integrated database were utilized. These fundamental parameters enabled the precise calculation of molar heat capacity at any given temperature T, in accordance with Equation (2).

The enthalpy of a substance at a temperature T, deviating from the standard reference of 298 K, was computed as follows:

(2) $$H_{T}=H_{298}+\int_{298}^{T}c_{\rho }dT+\sum H_{transition}$$

where $H_{298}$ denotes the standard enthalpy of the substance; $C_{\rho }\;$ rhorepresents the molar heat capacity; and $\sum H_{F}\;$ accounts for the enthalpy of phase transitions, including polymorphic transformations, fusion, and vaporization.

Entropy was evaluated through the following expression:

(3) $$S_{T}=S_{298}+\int_{298}^{T}\frac{c_{\rho }}{T}dT+\sum \frac{H_{transition}}{T_{transition}}$$

where $S_{298}\;$ is the standard entropy of the substance; $C_{\rho }$ is the molar heat capacity; and $\sum H_{F}/T$ represents the entropy contributions of phase transitions, encompassing polymorphic alterations, melting, and evaporation [43]. These refined formulations provide a robust theoretical foundation for assessing thermodynamic transformations within the studied system.

To assess the energy efficiency of the microwave treatment, the specific energy consumption was calculated for the “ENERGY K-50” system. The microwave generator was set to a power of 20 kW, and the clinker sample (volume of 0.001 m^3^ or 1 L) was processed for 5–7 min (an average of 6 min or 360 s) to achieve the desired phase transformations, such as the conversion of sphalerite (ZnS) to zincite (ZnO). This duration was determined based on preliminary trials to ensure complete phase transformation while minimizing thermal gradients. The total energy consumption (E) for one cycle was calculated as follows:

$$E=P\cdot t=20\times 10^{3}\;W\cdot 360\;s=7.2\times 10^{6}\;J=7.2\;\mathrm{MJ}$$

Given the clinker density of 4100 kg/m^3^, the mass (m) of the processed clinker per cycle was as follows:

$$m=\rho \cdot V=\frac{4100\;\mathrm{kg}}{m^{3}}\cdot 0.001{\;m}^{3}=4.1\;\mathrm{kg}$$

Thus, the specific energy consumption per kilogram of clinker was as follows:

$$E_{specific}=\frac{E}{m}=\frac{7.2\times 10^{6}}{4.1}\approx 1.76\times \frac{10^{6}\;J}{\mathrm{kg}}=1.76\;\mathrm{MJ}/\mathrm{kg}$$

This value can be compared to conventional pyrometallurgical methods, such as the Waelz process, which typically requires 3–5 MJ/kg for zinc recovery [44]. However, while microwave treatment offers lower energy consumption, it requires further research and optimization to ensure uniform heating and stable performance at an industrial scale.

## 3. Results and Discussion

### 3.1. Phase Analysis of the Initial Clinker

X-ray phase analysis revealed that the clinker is predominantly composed of quartz (19.8%) and magnetite (15.1%), with notable contributions from feldspar (anorthite, 10.4%) and troilite (10.3%). Other significant phases include chalcopyrite (8.4%), gypsum (8.2%), goethite (8.1%), lead oxide sulfate (6.1%), barium sulfate (4.6%), sphalerite (4.2%), magnesite (3.6%), and marmatite (2.4%). The clinker contains 3.21% copper, comparable to the 3.47% zinc content (Table 1). While this suggests potential as a copper source, the study prioritized zinc due to its prevalence in refractory phases (sphalerite and marmatite). Future work could explore co-extraction of copper, particularly as microwave treatment also forms copper oxides (CuO/Cu_2_O, 1.5%, Table 2), which are amenable to acid leaching. X-ray diffraction (XRD) analysis (Table 2) revealed significant phase transformations in the clinker after microwave treatment. Zinc-bearing phases (sphalerite, 4.2%; marmatite, 2.4%) are completely converted to zincite (ZnO, 6.6%), while other phases, such as troilite and goethite, decrease, and new phases, including hematite (12.4%) and anhydrite (3.5%), form, enhancing the clinker’s leachability. A comprehensive overview of these phases and their changes is provided in Table 2.

### 3.2. Effects of Microwave Irradiation on Phase Transformations and Thermal Stress in Clinker

Microwave treatment was 915 MHz. Microwaves heat the material through dielectric losses, described by the following heating power equation:

(4) $$P=2\pi f\varepsilon_{0}\varepsilon^{\prime \prime }E^{2}V$$

where (P) is the heating power (W), $f=915\times 10^{6}\;\mathrm{Hz}$ is the microwave frequency, $\varepsilon_{0}$ = 8.854 × 10^−12^ F/m is the permittivity of free space, $\varepsilon^{\prime \prime }=\varepsilon_{r}tan\delta \approx 5$ is the imaginary part of the dielectric permittivity of the clinker (considering magnetite), E = 1000 V/m is the electric field strength, and V = 0.001 m^3^ is the sample volume. For these parameters, P≈ 254 W, enabling the clinker to be heated to 600 °C in 5–7 min.

The penetration depth of microwaves into the material determines the uniformity of heating and is calculated as follows:

(5) $$D_{p}=\frac{\lambda_{0}}{2\pi \sqrt{2\varepsilon^{\prime }(\sqrt{1+\left(\frac{\varepsilon^{\prime \prime }}{\varepsilon^{\prime }}{)}^{2}-1\right)}}}$$

where $D_{p}$ is the penetration depth (m), $\lambda_{0}=\frac{c}{f}$ is the wavelength in vacuum, c = 3 × 10^8^ M/C, $\varepsilon^{\prime }\approx 10,\;\mathrm{and}\;\varepsilon^{\prime \prime }\approx 5$. For $f=915\times 10^{6}\mathrm{Hz}$, $D_{p}\approx 0.03\;m$. This indicates that microwaves penetrate the clinker to a depth of several centimeters, sufficient to heat particles sized 0.5–1 cm, but lead to non-uniform heating due to differences in $\varepsilon^{\prime \prime }$ among minerals [45].

**Figure 4:**
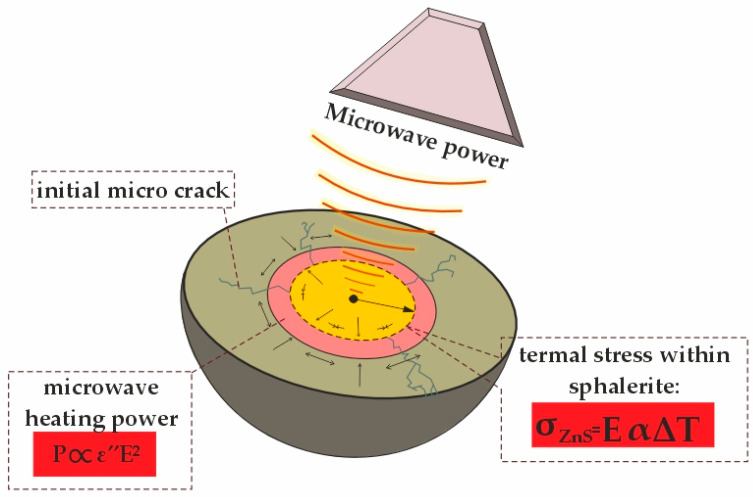
Microwave impact on sphalerite and thermal stress distribution.

Heat from microwaves propagates slowly in sphalerite due to its low thermal conductivity. The temperature increase was calculated as follows:

(6) $$\Delta T=\frac{P\cdot t}{\rho \cdot c_{p}\cdot V}\;$$

where P = 254 W, t = 300 s (5 min), ρ = 4100 kg/m^3^ is the density of sphalerite, $c_{p}$ = 480 J/(kg·K) is the specific heat capacity, and *V* = 0.001 m^3^ is the sample volume, yielding ΔT≈38 °C. However, due to the low thermal conductivity of sphalerite (k = 2.5 W/(m·K) and variations in microwave absorption (magnetite heats more intensely than sphalerite), localized temperature gradients arise, leading to cracking (see Figure 4).

Non-uniform heating induces thermal stress in sphalerite, calculated as follows:

(7) $$\sigma =E\alpha \Delta T{\left(\frac{r_{local}}{r_{total}}\right)}^{2}\;$$

where E = 75 × 10^9^ Pa is the modulus of elasticity of sphalerite, α = 6.5 × 10^−6^ K^−1^ is the thermal expansion coefficient, ΔT = 575 K is the local temperature difference between minerals, and $r_{local}/\;r_{total}$= 0.1. This yields σ≈ 2.8 MPa, which promotes cracking (see Figure 4).

This cracking along with the phase transformation of ZnS into ZnO (Table 3) account for the increase in zinc extraction efficiency from 39.1% to 92.5% following microwave treatment.

Microwave treatment at 600 °C induces phase transformations in the clinker, primarily affecting zinc-bearing phases: sphalerite (ZnS, 4.2%) and marmatite (Zn_0.66_Fe_0.34_S, 2.4%) oxidize to zincite (ZnO). After 3–4 min, the surface exhibits a reduction in sulfur content to 0.5% and the presence of oxygen (23%), indicating partial oxidation, as confirmed by energy-dispersive spectroscopy (EDS). Extending the treatment to 5–7 min results in complete transformation, with oxygen content reaching 22.94% and zinc content stabilizing at 77.06%, signifying near-complete conversion to ZnO. The phase composition changes in the clinker before and after microwave treatment under optimal conditions are summarized in Table 2.

**Figure 5:**
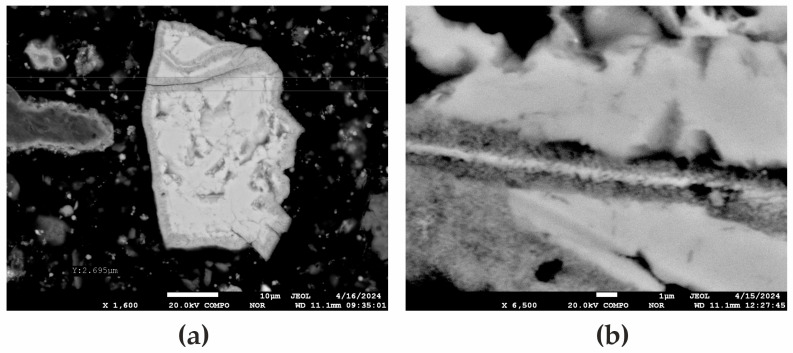
SEM images of zincite after microwave treatment at 600 °C for 3–4 min: (**a**) magnification ×1600, scale bar 10 µm; (**b**) magnification ×6900, scale bar 1 µm.

Physical changes, such as microcracks in zincite due to thermal stresses, enhance porosity, as shown in the SEM images (Figure 5). These microcracks increase reactivity, contributing to the improved zinc extraction efficiency.

A study by [46] showed that conventional roasting of sphalerite at 600 °C for 90 min achieves a maximum reaction rate of 1.23 × 10^−3^ min^−1^. Microwave roasting reduces the treatment time to 5–7 min at the same temperature, efficiently converting zinc-bearing phases into ZnO via the following reaction:

ZnS + 1½O_2_ → ZnO + SO_2_ (8)

Thermodynamic analysis confirms that this exothermic reaction involves the cleavage of ZnS bonds and the formation of more stable ZnO and SO_2_ gas, classified as a first-order reaction (Table 3).

**Figure 6:**
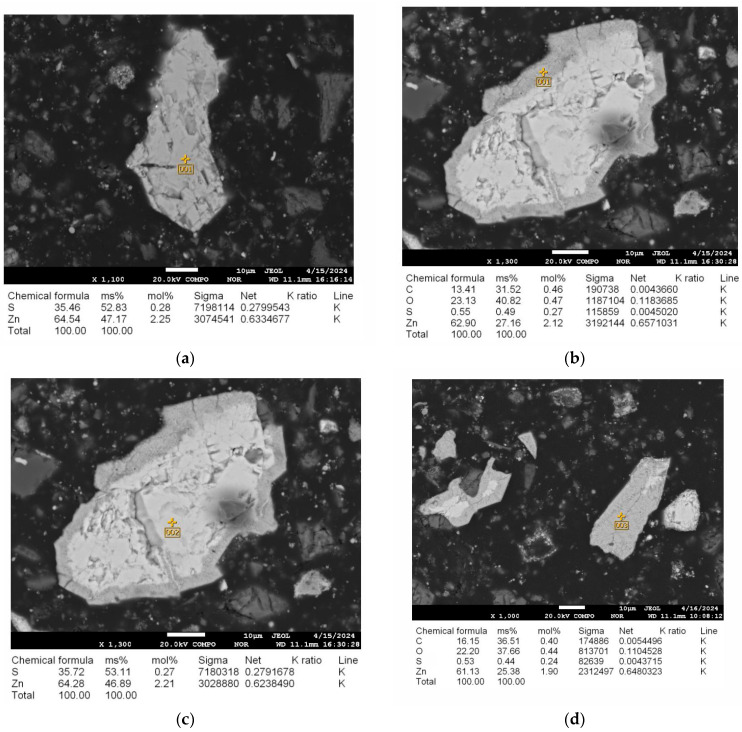
(**a**) Microstructure and EDS analysis of sphalerite before microwave treatment at 25 °C; (**b**,**c**) Microstructure and EDS analysis of sphalerite after microwave treatment at 600 °C for 3–4 min; (**d**) Microstructure and EDS analysis of sphalerite after microwave treatment at 600 °C for 5–7 min.

The EDS analysis confirms these transformations quantitatively. Before microwave treatment, sphalerite exhibits a homogeneous composition with 35.46% sulfur and 64.54% zinc (Figure 6a). After 3–4 min of treatment at 600 °C, the surface shows a significant reduction in sulfur to 0.55% and the presence of oxygen at 23.13%, while the zinc content is 64.28%, indicating partial oxidation to ZnO (Figure 6b). The inner layers retain a nearly unchanged composition, with 35.72% sulfur and 64.28% zinc (Figure 6c). After 5–7 min of treatment, the transformation is complete, with the oxygen content reaching 22.94% and the zinc content stabilizing at 77.06%, confirming the formation of zinc oxide (Figure 6d).

The presence of carbon in the analyzed samples (Figure 6b) results from carbon deposition caused by the electron beam during microprobe analysis, with the carbon tape used for sample preparation serving as the carbon source [47,48].

### 3.3. Leaching Experiments

To evaluate the efficiency of acid leaching of zinc from clinker, experiments were conducted under atmospheric conditions, varying key technological parameters, including the solid-to-liquid ratio (S:L), sulfuric acid concentration, and treatment duration. The effect of the S:L ratio on zinc dissolution was analyzed based on the potential-pH (φ–pH) diagram of the Zn–Fe–H_2_O system.

A temperature of 25 °C was selected to minimize energy consumption for potential industrial-scale applications, making the process economically viable. A stirring speed of 300 rpm was used as the optimal value, as previous studies demonstrated that this parameter yields the highest zinc extraction efficiency. Consequently, the influence of stirring speed was not investigated in this study.

**Figure 7:**
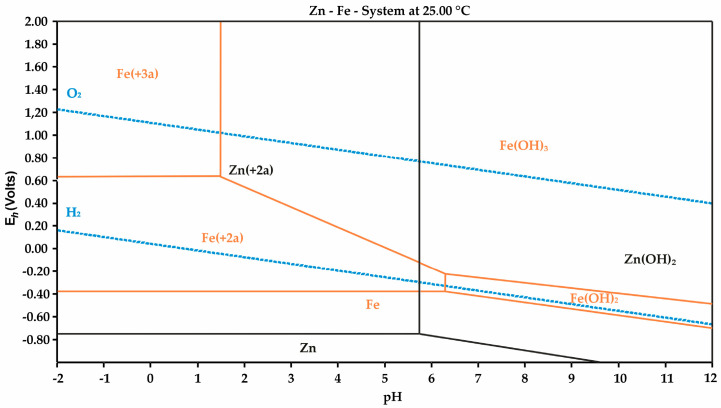
Potential–pH diagram for the Zn–Fe–H_2_O system at 25 °C (Me^n+^ = 0.01, 25 °C, 1.0 bar).

Increasing the S:L ratio leads to a higher overall content of the leaching agent, which enhances iron dissolution and increases the concentration of Fe ions in the solution, affecting subsequent processing stages. To assess these processes, a potential-pH (φ–pH) diagram of the Zn–Fe–H_2_O system was constructed at a concentration of Me^n+^ = 0.01 mol/dm^3^, a temperature of 25 °C, and a pressure of 1.0 bar (Figure 7).

The experimental data revealed the following dependence of the filtrate pH on the S:L ratio: at S:L = 3, pH ≥ 4; at S:L = 4, pH = 1.0–1.5; and at S:L = 5, pH ≤ 0.5. According to the diagram (Figure 7), Fe^3+^ ions begin to precipitate as Fe(OH)_3_ at pH ≥ 2.0, consistent with previous studies [49,50,51,52]. At pH values above 2, the formation of Fe(OH)_3_ is accompanied by the sorption of free Zn^2+^ ions, leading to zinc losses due to co-precipitation with iron hydroxide.

Thus, optimizing the S:L ratio is a critical factor in enhancing zinc extraction efficiency and minimizing solution contamination with iron. Based on the obtained data, an S:L ratio of 1:4 was selected for subsequent experiments, ensuring minimal Fe ion concentration while maintaining high selectivity for zinc leaching.

**Figure 8:**
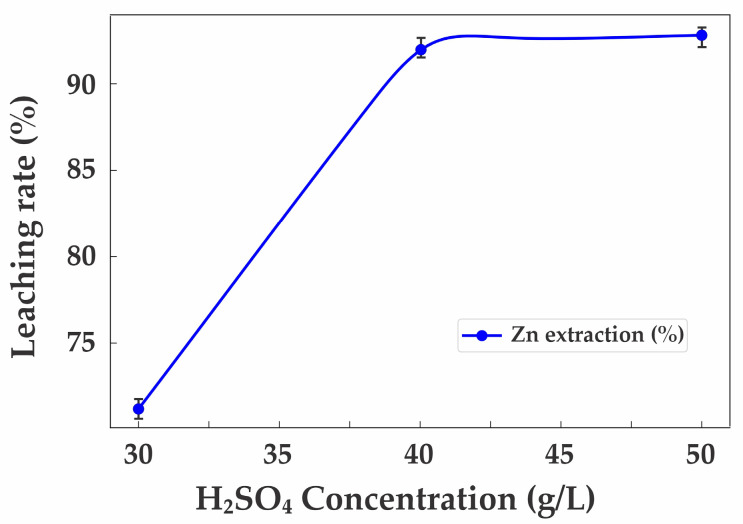
Effect of sulfuric acid concentration (g/L) on zinc extraction efficiency.

The selection of optimal sulfuric acid concentration and process duration also plays a decisive role in maximizing zinc extraction efficiency (Figure 8).

This study investigated the effect of H_2_SO_4_ concentration in the range of 20–60 g/L and leaching duration from 2 to 8 h. Analysis of the obtained data showed that, at a concentration of 30 g/L, the zinc extraction efficiency was 71.2%; at 40 g/L, it reached 92.5%; and at 50 g/L, it was 92.8%, indicating no significant improvement with further increases in acid concentration. This is attributed to the intensified dissolution of impurity elements, such as Fe, at concentrations above 50 g/L, which can negatively affect the purity of the resulting solution.

**Figure 9:**
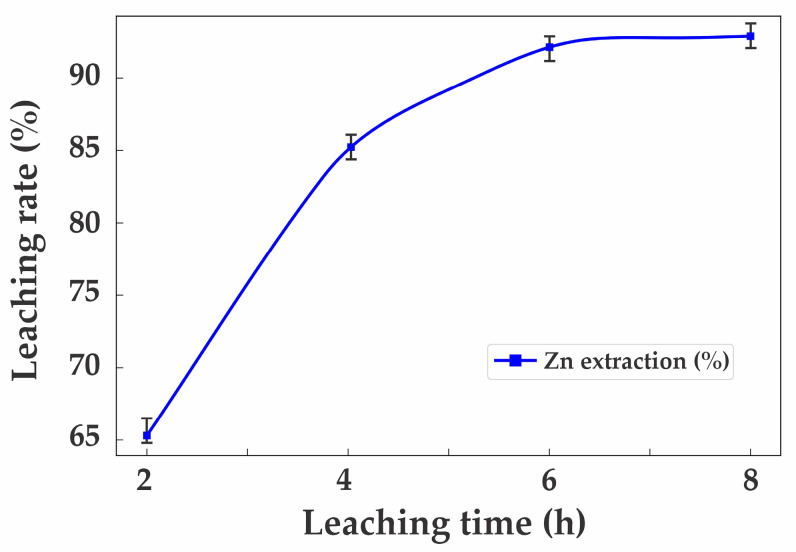
Effect of leaching duration on zinc extraction efficiency.

Similarly, the effect of leaching duration is shown in Figure 9.

At 2 h, the extraction efficiency was 65.3%; at 4 h, it increased to 85.6%; at 6 h, it reached 92.5%; and further extending the duration to 8 h resulted in only a marginal increase to 92.9%. Consequently, the optimal leaching parameters were established as 40 g/L H_2_SO_4_ and 6 h.

### 3.4. Comparison of Conventional and Microwave-Assisted Leaching

**Figure 10:**
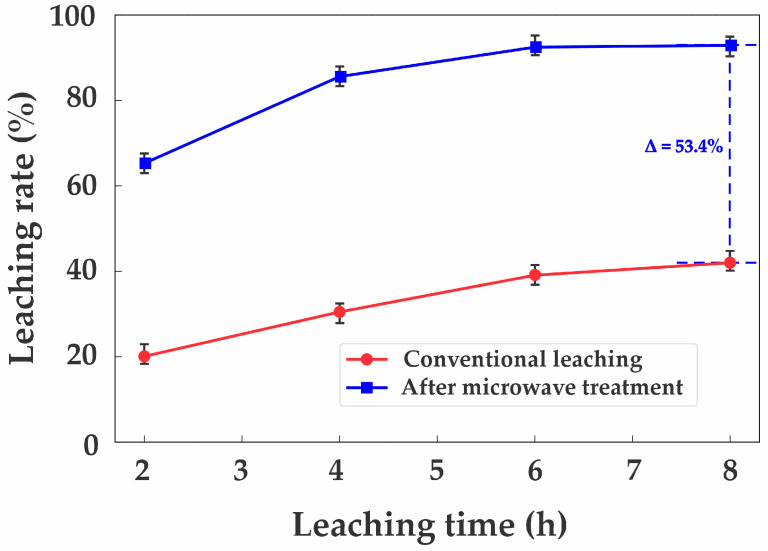
Comparison of conventional and microwave-assisted zinc leaching.

To evaluate the effectiveness of microwave treatment, a comparison was conducted between conventional acid leaching and the process with prior microwave exposure. Under identical technological parameters (S:L ratio, acid concentration, stirring speed, and duration), the zinc extraction efficiency after microwave treatment reached 92.5%, compared to only 39.1% without pre-treatment, as shown in Figure 10.

This effect is attributed to the microwave treatment facilitating the near-complete conversion of sphalerite (ZnS) into the oxide form, zincite (ZnO), which exhibits significantly higher reactivity during sulfuric acid dissolution.

For a more detailed understanding of the differences in the solubility of zinc phases, a thermodynamic analysis of the leaching reactions was performed. The data presented below elucidate the advantage of microwave treatment due to the transformation of ZnS into ZnO, which possesses higher reactivity in sulfuric acid.

As shown in Table 3, zinc oxide (ZnO) exhibits significantly higher reactivity compared to zinc sulfide (ZnS) when interacting with sulfuric acid at 298 K. This is evidenced by the substantially more negative Gibbs free energy of the reaction (ΔG = −97.817 kJ) for ZnO, indicating its high thermodynamic favorability and near-irreversibility. In contrast, the dissolution reaction of ZnS has a less negative ΔG (−59.498 kJ), suggesting a lower tendency for spontaneous progression.

Additionally, the difference in thermodynamic characteristics is reflected in the equilibrium constant (K): for ZnO, it is 1.376 × 10^17^, seven orders of magnitude higher than for ZnS (2.659 × 10^10^), indicating a significantly greater shift of equilibrium toward the reaction products. Furthermore, the enthalpy of the ZnO dissolution reaction (ΔH = −103.665 kJ) is negative, confirming its exothermic nature, whereas ZnS dissolution (ΔH = 148.283 kJ) requires substantial heat input, hindering the process. Thus, the high reactivity of ZnO in sulfuric acid is driven by more favorable thermodynamic conditions, while ZnS dissolution necessitates additional oxidation, making it less efficient for direct acid leaching.

## 4. Conclusions

This study confirms the efficacy of integrating microwave thermal treatment with acid leaching to enhance zinc recovery from refractory clinker. Microwave irradiation induces phase transformations, converting sphalerite (ZnS) into zincite (ZnO), which significantly improves chemical reactivity and increases zinc extraction from 39.1% (without treatment) to 92.5% under optimized conditions. These findings, supported by thermodynamic analysis, highlight the potential of microwave pre-treatment to intensify hydrometallurgical processes for secondary zinc resources. While microwave treatment demonstrates lower energy consumption (1.76 MJ/kg) compared to conventional pyrometallurgical methods (3–5 MJ/kg), its economic viability depends on capital costs for microwave equipment, operational scale, and zinc market prices. Preliminary estimates suggest that the energy cost for microwave treatment is approximately $0.05–0.10 USD/kg of clinker (based on industrial electricity rates of 0.10 USD/kWh), which is competitive given zinc prices of ~2.5–3.0 USD/kg (as of 2025). However, a comprehensive techno-economic analysis is needed to confirm scalability and cost-effectiveness, which we propose as a focus for future research. The method offers energy-efficient processing and reduced environmental impact, making it a promising approach for industrial applications. Future research should prioritize techno-economic assessments to evaluate scalability, investigate the use of alternative leaching agents to minimize acid consumption, and employ process modeling to optimize operational parameters for large-scale implementation.

## Figures and Tables

**Table 1:** Elemental composition of clinker based on X-ray fluorescence analysis.

Elemental Content, %									
**O**	**Mg**	**Al**	**Si**	**P**	**S**	**K**	**Ca**	**Ti**	**Cr**
31.268	0.752	3.062	9.592	0.075	4.427	0.320	5.268	0.403	0.057
**Mn**	**Fe**	**Ni**	**Cu**	**Zn**	**As**	**Sr**	**Mo**	**Ba**	**Pb**
1.191	20.980	0.023	3.207	3.468	0.121	0.077	0.015	1.855	2.579

**Table 2:** Phase compositions of the clinker samples before and after microwave treatment under optimal conditions.

Name	Content, %	
	Before Microwave Treatment	After Microwave Treatment
Quartz SiO_2_	19.8	19.5
Magnetite Fe_3_O_4_	15.1	15.1
Feldspar (Anorthite) CaAl_2_Si_2_O_8_	10.4	10.5
Troilite FeS	10.3	4.5
Chalcopyrite CuFeS_2_	8.4	6.5
Gypsum CaSO_4_·2H_2_O	8.2	4.5
Goethite FeO(OH)	8.1	1.5
Lead Oxide Sulfate Pb_3_O_2_SO_4_	6.1	5.3
Barium Sulfate BaSO_4_	4.6	4.8
Sphalerite ZnS	4.2	-
Magnesite MgCO_3_	3.6	2.3
Marmatite Zn_0.66_Fe_0.34_S	2.4	-
Hematite Fe_2_O_3_	-	12.4
Zincite ZnO	-	6.6
Anhydrite CaSO_4_	-	3.5
Copper Oxides CuO/Cu_2_O	-	1.5
Magnesium Oxide MgO	-	1.3
Lead Oxide PbO	-	0.8

**Table 3:** Thermodynamic parameters of zinc leaching reactions with sulfuric acid.

Reaction	T, K	ΔH, kJ	ΔS, J/K	ΔG, kJ	K	Log K
ZnS(s) + 4H_2_SO_4_(aq) **→** ZnSO_4_(aq) + 4SO_2_(g) + 4H_2_O(l)	298	148.283	696.902	−59.498	2.659 × 10^10^	10.425
ZnO(s) +H_2_SO_4_(aq) **→** ZnSO_4_(aq) +H_2_O(l)		−103.665	−19.614	−97.817	1.376 × 10^17^	17.139

## Data Availability

The original contributions presented in this study are included in the article. Further inquiries can be directed to the corresponding author.
